# Urban water network upgrades improve quality and access to drinking water in the PAASIM matched cohort study in Beira, Mozambique

**DOI:** 10.21203/rs.3.rs-6313590/v1

**Published:** 2025-04-16

**Authors:** Courtney Victor, Joshua Garn, Rassul Nalá, João Manuel, Magalhães Mangamela, Sandra McGunegill, Jedidiah Snyder, Sydney Hubbard, Christine Fagnant-Sperati, Joe Brown, Thomas Clasen, Konstantinos Konstantinidis, Elizabeth Rogawski McQuade, Lance Waller, Karen Levy, Matthew Freeman

**Affiliations:** Emory University; University of Nevada, Reno; Instituto Nacional de Saúde; Instituto Nacional de Saúde; Autoridade Reguladora de Água e Saneamento (AURA); Emory University; Emory University; The Aquaya Institute; University of Washington; UNC Chapel Hill; Emory University; Georgia Institute of Technology; Emory University; Emory University; University of Washington; Emory University

## Abstract

Rigorous evaluations of community water supply interventions are necessary to understand their impact on water quality and access. Our study in Beira, Mozambique assessed the impact of water infrastructure improvements in neighborhoods with or without piped water network upgrades. Data were collected from 642 households on microbial contamination in stored and source water, water access, and satisfaction with water service. The intervention reduced source water *E. coli* contamination by 33% and stored water by 14%. Regardless of intervention status, having a direct connection to the piped water network (versus none) was associated with 24% lower *E. coli* in source water but no difference in stored water. Intervention households and households with a direct connection had better water access and higher satisfaction. These findings suggest that urban water supply interventions can improve access to a safe water supply, but improvements may be compromised by water storage practices due to water intermittency.

Access to a continuous safe water supply is crucial for waterborne disease prevention, facilitation of hygiene practices, economic productivity, and overall well-being [1–3]. An estimated 1.4 million deaths could be prevented annually by providing adequate safe water, sanitation, and hygiene (WaSH) services [3]. Sustainable Development Goal 6.1 calls for universal access to safely managed water services *(i.e.*, water provided at the home that is free from chemical and microbial contamination) [4]. In 2022, 37.9% of the population of low- and middle-income countries (LMICs) and 8.8% of the population in sub-Saharan Africa were estimated to have access to safely managed drinking water [3].

Evidence on the impact of community-level improvements to piped water supply on water quality and access is needed to guide planning improvements to WaSH service delivery under conditions of inadequate resources [5]. There have been no rigorous impact evaluations of piped water service delivery improvements on water quality and access in urban, low-income settings, despite considerable investment in improving urban water systems. Only 11 studies have assessed provision of piped water to the premises [6–16] (Supplemental Fig. 1). Of these, 6 studies included a water quality assessment, while the majority (n = 10) focused on improving health outcomes. Only two interventions had a goal of improving quality of water piped to the premises [11, 13] and only one of these was in an urban setting [13]. Evidence from these two studies suggests provision of an improved drinking water supply on the premises with higher water quality is associated with reductions in diarrhea [17]. None of these studies evaluated an urban, community-level water infrastructure improvement project. Understanding the impact of community-level urban water investments on water quality and access, and health outcomes, would allow for improved investment programming.

Water quality is a critical exposure on the causal pathway between a water intervention and health outcomes, but very few community water supply interventions have assessed changes in exposure to fecal contamination targeted by the interventions under study [18]. A water supply intervention theoretically prevents disease if it: a) is capable of reducing enteric pathogen exposure, (b) is introduced into a vulnerable population, (c) achieves high coverage, (d) ensures correct and consistent use, and (e) reduces population exposure to enteric pathogens [18]. Assessment of an intervention’s impact on water quality can provide insights into why an intervention was or was not able to achieve health gains [18]. There have been recent calls for WaSH studies that explicitly and rigorously measure the impact of interventions on reducing harmful exposures [18].

To maximize health gains, water must not only be microbially safe, but also continuously delivered in sufficient quantities. Yet, safe drinking water access in epidemiological studies is often defined solely by microbial contamination. A key component of the effectiveness of a piped water supply improvement is the level of service defined by the household experience of the intervention over time. The household’s perspective of the service, affordability, availability, and quality impacts their water consumption and management practices[19].In Mozambique, we previously found that increased intermittency was associated with lower satisfaction with water quality, pressure, and service [20]. A comprehensive assessment of water quality, access, and the consumer-perspective of water service can contribute important information for researchers when assessing the impact of these interventions and to stakeholders who are planning improvements to piped water supply.

Here we provide evidence on the impact of urban piped water service delivery improvements [17] on intermediate exposure outcomes, through a matched control trial of the effects of community-level water supply improvements on water quality and access. This analysis includes an evaluation of water quality and access as a distinct exposure assessment that complements the health outcomes of the impact evaluation [21], reported elsewhere. We test whether living in an intervention neighborhood, which received piped water service delivery upgrades, impacted source water contamination (primary outcome), stored water contamination, water availability, affordability, and accessibility (additional outcomes), and assess associations between having a direct connection and these outcomes throughout five different household visits. We hypothesize that living in intervention neighborhoods and/or having a direct connection to an improved water supply led to improvements in water quality and access.

## Results

**Figures 1:**
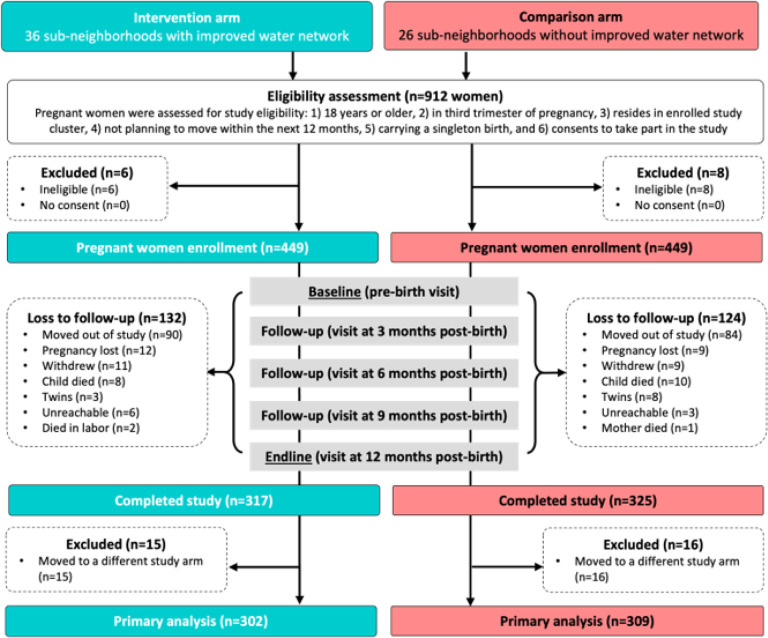
Cohort profile

We enrolled 898 pregnant women into the PAASIM study, and 642 (71%) completed the study, leading to a total of 317 intervention households and 325 comparison households with complete data after 12 months of follow-up (see ‘Data sources’ in Methods) (Fig. 1). Households that moved to a neighborhood with a different intervention designation were excluded from the analyses of the impact of the intervention *(n* = 31), so our final intervention dataset included 302 intervention and 309 comparison households.

### Intervention fidelity

At the 12-month visit, 139/302 (46%) of intervention households were connected to the water network, compared to 110/309 (36%) in comparison households, representing 34% more connections (aRR 1.34, 95% CI 1.05–1.72) (Supplemental Table 1).

### Characteristics of households at enrollment

At enrollment, intervention households had lived in the residence for longer, had lower fixed employment of the primary wage earner, and higher prevalence of primary caregivers with secondary education than comparison households (Table 1). Prevalence of high poverty and basic sanitation access were similar between arms. Households with a direct connection to the piped water network had lived in the residence for longer, higher basic sanitation access, higher prevalence of primary caregivers with secondary education, higher prevalence of primary wage earner fixed employment, lower poverty, and lower prevalence of household or yard flooding in the last month compared to households without a direct connection (Table 1).

### Impact of the intervention on water quality and access

**Figures 2:**
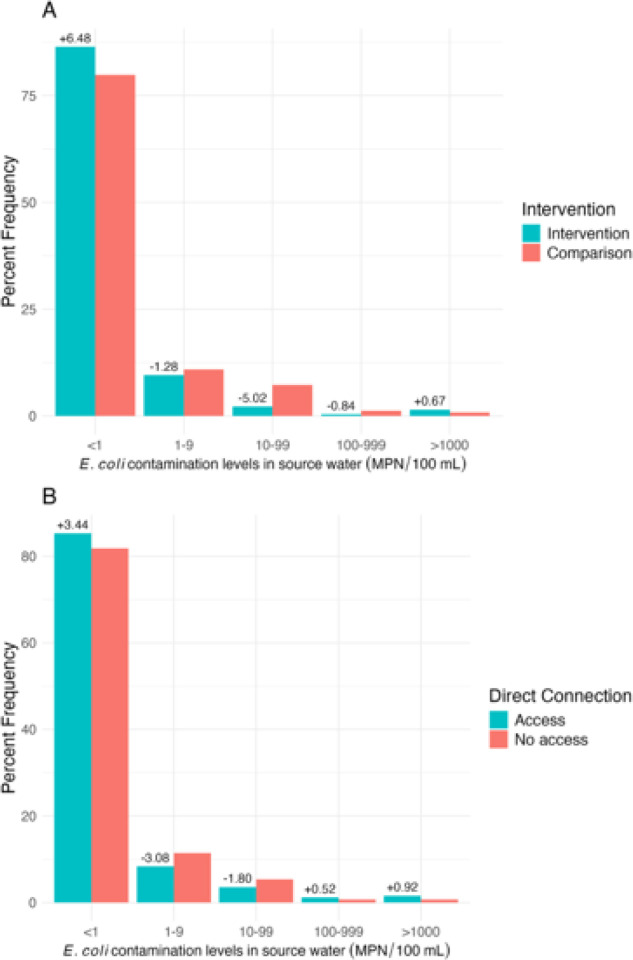
Percent frequency of *E. coli* contamination levels in source water at 12-months by A) sub-neighborhood intervention status and B) direct connection status. The ‘+’ and ‘-’ above the columns indicate the difference in percent frequency between intervention and control (A) or access and no access (B).

At the end of follow-up (12 months of age), the prevalence of *E. coli* in source water (primary outcome) was 33% lower in intervention households than comparison households (aPR = 0.67, 95% CI: 0.46, 0.98) (Table 2). Frequency of safe water (< 1 MPN *E. coli* /100mL) was higher in intervention households versus comparison households and lower for categories of 1–9, 10–99, and 100–999 MPN/100 mL (Fig. 2A). Only 13% of samples from intervention households and 20% of samples from comparison households had any detectable *E. coli* at the end of follow-up (Table 2).

**Figures 3:**
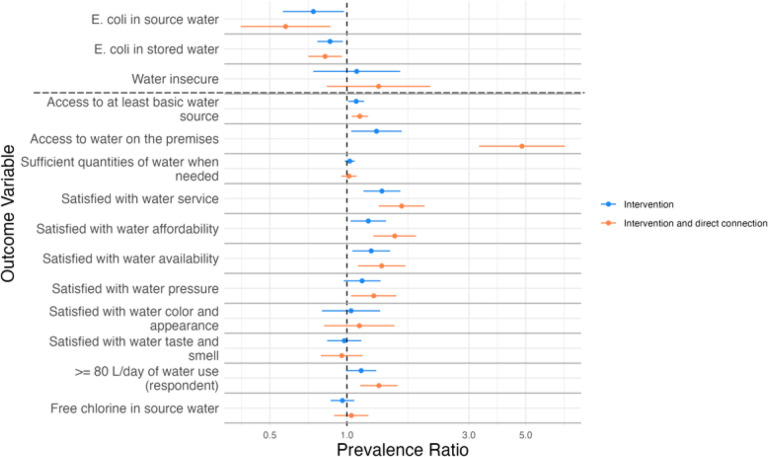
Impact of the intervention and joint effect of the intervention and having a direct connection on water quality and access. We hypothesize to observe a prevalence ratio <1.0 for *E. coli* in source water, *E. coli* in stored water, and water insecure and a prevalence ratio >1.0 for all other variables. The distinction is indicated with a dashed horizontal line.

Across all timepoints, intervention households had 26% lower prevalence of *E. coli* in source water (aPR = 0.74, 95% CI: 0.56, 0.97), and 14% lower prevalence of *E. coli* in stored water (aPR = 0.86, 95% CI: 0.77, 0.96) versus comparison households (Fig. 3, Table 2). The intervention households had an increased prevalence of access to at least basic water defined by the Joint Monitoring Programme [22] (aPR = 1.09, 95% CI: 1.01, 1.17), water available on the premises (aPR = 1.31, 95% CI: 1.04, 1.64), and satisfaction with water service (aPR = 1.37, 95% CI: 1.16, 1.62), water affordability (aPR = 1.21, 95% CI: 1.03, 1.42), and water availability (aPR = 1.24, 95% CI: 1.05, 1.48) compared to comparison households. The prevalence of water insecurity was 22% among intervention households and 16% among comparison households (aPR = 1.09, 95% CI: 0.74, 1.62) (Fig. 3, Table 2). There was little difference in other quality and access variables between the intervention and comparison households, including in the prevalence of safe free chlorine levels in source water, access to sufficient quantities of water, and satisfaction with water taste and smell. These results are comparable to the analysis of the impact of the intervention at the end of follow-up alone (Supplemental Table 2).

### Association between having a direct connection and water quality and access

Among the 642 households that completed the study, 262 had a direct connection and 380 did not. The prevalence of *E. coli* in source water (primary outcome) was 15% among households with a direct connection and 18% among households without a direct connection (aPR = 0.76, 95% CI: 0.53, 1.10) (Table 3). We observed higher frequency of safe water (< 1 MPN *E. coli*/100ml) in households with a direct connection compared to households without a direct connection, and lower frequency of higher contamination categories (Fig. 2B).

Across all timepoints, our data revealed a modest, though imprecise, protective association between having a direct connection and *E. coli* in source water (12% vs 15%; aPR = 0.80, 95% CI 0.59, 1.08) (Table 3). Data from households with and without a direct connection revealed similar prevalence of *E. coli* in stored water (46% vs 45%; aPR = 1.00, 95% CI: 0.89, 1.11). Having a direct connection was associated with a higher prevalence of access to at least basic water (aPR = 1.04, 95% CI: 1.01, 1.07), safe source water free chlorine levels (aPR = 1.13, 95% CI: 1.02, 1.26), water consumption (aPR = 1.31, 95% CI: 1.13, 1.53), and satisfaction with water service (aPR = 1.19, 95% CI: 1.04, 1.36), affordability (aPR = 1.31, 95% CI: 1.17, 1.46), pressure (aPR = 1.15, 95% CI: 1.02, 1.29), and appearance (aPR = 1.15, 95% CI: 0.97, 1.38) compared to households without a direct connection. Having a direct connection was associated with a higher prevalence of water insecurity (aPR = 1.27, 95% CI: 1.03, 1.58) compared to households without a direct connection. There was no association between having a direct connection and satisfaction with water taste and smell (aPR = 1.00, 95% CI: 0.888, 1.13) or having sufficient quantities of water (aPR = 1.00, 95% CI: 0.95, 1.04). These results were comparable to the analysis of the association between having a direct connection and water quality and access at the end of follow-up alone (Supplemental Table 3).

### Joint effect of living in intervention neighborhoods and direct connection

We found significant interaction between intervention status and having a direct connection on satisfaction with water service (*p*-value = 0.05) (Fig. 3), but no other water quality and access variables (Supplemental Table 5). Living in intervention households with a direct connection was associated with an increase in prevalence of always being satisfied with water service compared to living in comparison households with no direct connection (aPR = 1.64, 95% CI: 1.33, 2.01). Imbalances found between groups we used for controlling variables in this analysis are in Supplemental Table 4.

## Discussion

To our knowledge, PAASIM is the first evaluation of urban piped water supply infrastructural improvements to focus on intermediary water quality and access variables as independent outcomes. We found that the intervention led to lower prevalence of source water *E. coli* contamination, and increased water access and satisfaction. Having a direct connection was associated with lower prevalence of *E. coli* contamination in source water, but not stored water, and was associated with an increase in safe source water free chlorine levels. Source water contamination was low across study arms but elevated in stored water samples.

Though we found that intervention households had a lower prevalence of stored water contamination, having a direct connection was not protective against stored water contamination. Water service was intermittent, with average availability of 13 hours/day reported by participants. Likely because water was not continuously available, 99% of respondents reported storing their water. Prevalence of *E. coli* in source water ranged from 12–15%, while prevalence of *E. coli* in stored water ranged from 44–48% (Table 2). This aligns with research documenting that stored water is more likely to be contaminated than water obtained directly from the source [23, 24]. Additionally, provision of a continuous water supply, which is not specified in SDG 6.2, is critical for maintaining microbially-safe water [25, 26]. Other evaluations of piped water interventions on premises also reported continuing water storage after the intervention [7, 9, 14–16], even when continuous access was provided [9]. Despite results indicating that higher quality source water was delivered to intervention households and households with direct connections, continued water storage may minimize potential health gains of upgraded water supply.

Our findings also suggest that the intervention led to higher access to water on the premises, and access to at least basic water was higher among intervention households than comparison households. We found that the intervention and having a direct connection resulted in higher satisfaction with water service, availability, and affordability. Moreover, intervention households with a direct connection reported having the highest satisfaction with water service. While these variables may be indirectly related to health, satisfaction with water services is important to overall quality of life and can influence uptake of interventions. Access to a safe water supply on the premises has been associated with overall well-being and other positive outcomes [27], particularly for women and girls who often bear the burden of water collection [28]. Capturing the household experience of water service delivery can provide important information about the additional benefits provided by these interventions [29].

Our results indicate that living in a community that has received piped water supply improvements may lead to an increased access to water on the premises, though coverage was not universal. One of the priorities of the water utility was to increase direct connections in the intervention neighborhoods. The intervention led to a 34% increase in direct connections, but coverage was still incomplete; 54% of intervention households did not have a direct connection at the end of follow-up.

For those without a direct household connection, access to water is typically through public standpipes or purchasing from neighbors [30–32]. Using a public standpipe is typically more expensive than purchasing from neighbors and requires increased time to collect water [33]. While purchasing from neighbors can minimize time expenditures, it can be less reliable [33]. Research within informal settlements in Maputo, Mozambique found that having a direct connection was associated with decreased cost and water collection time expenditures compared to those access via public standpipe [30, 33, 34]. These factors have implications on mental health and well-being. Worry about water cost has been associated with increased anxiety [35]. Dissatisfaction with water services and worry about affordability or availability can predispose individuals to external stressors such as fear of disease, lost opportunities, and intra-household conflicts [36].

Consumer perception of water service and quality does not always align with monitored quality and service [37, 38]. Indeed, our measure of water quality (*E. coli* in source water) was not correlated with water taste and smell nor color and appearance (Supplemental Fig. 2). The consumer’s perspective of their water service and quality impacts their interaction and use of the service [19]. When evaluating a water supply system, it is critical to capture the functionality of the system and the household’s experience of effective service over time. Incorporating data on consumer satisfaction can help tailor interventions to provide higher quality service. Our findings indicate that living in intervention neighborhoods and having a direct connection resulted in improved operational sustainability of the water system, as measured by the household experience. Future studies which explore the misalignment between satisfaction and quality indicators could offer valuable insights for tailoring interventions based on consumers’ actual experiences.

We observed a pattern of increased water insecurity among intervention households and households with a direct connection versus comparison households and those without a direct connection. This result may be driven by a single item on the Household Water Insecurity Experiences (HWISE) survey regarding interruptions to service. Intervention households reported interruptions to their water service more frequently than comparison households. This aligns with a previous study that found interruption to water service to be the one of the most prevalent household water insecurity experiences in an informal settlement community in Colombia that relied on a recently upgraded piped water supply system [39]. The household’s location may also impact service interruption. Our previous study in Beira found that increased distance from the water main pipes was associated with greater water intermittency [20]. In the direct connection analysis, a larger proportion of respondents with a direct connection responded ‘Rarely’ experiencing insecurity for each question compared to households without a direct connection that more frequently responded ‘Never’ to each question. These findings may indicate that customers who are investing financial resources into water service may be more aware and critical of interruptions to service, despite reporting higher overall satisfaction with service. Additional qualitative data to contextualize the household water insecurity experience would aid local stakeholders in improving the reliability of the distribution system.

There were some limitations with our study. Due to the nature of the intervention, our research team had no control over implementation of the intervention, and we were unable to randomize households to intervention and comparison neighborhoods. Without randomization, there is a risk of selection bias and unmeasured confounding. This risk is more pronounced in the direct connection analysis, as the matched study design was implemented based on neighborhood intervention status. Water quality and access may also be more variable than what we captured at five timepoints. Survey questions related to satisfaction and access are subject to recall bias. Qualitative data on motivations for water storage practices and on the household water insecurity experience could provide important insight to guide future improvements to these interventions. Data from the water utility on service outages and intermittent access could complement our data by providing important context on the reliability of these systems. Our longitudinal study provided important insight into changing quality and access over time and could be complemented by studies on the longevity and sustainability of this and other piped water supply interventions in urban, low-income settings.

## Conclusion

To our knowledge, this study is the first evaluation of a piped water supply intervention to utilize a comprehensive assessment of water quality, accessibility, and consumer-satisfaction with water services, which provides insight into water quality and access that is often missing from impact evaluations. We provide evidence to suggest that expansion of water services to low-income neighborhoods, though expensive, can improve water quality and increase water access and satisfaction. Our findings highlight the importance of investing in community water supply interventions and of expanding direct connections to these services. We did not find a similar impact of having a direct connection on stored water quality and found an opposite effect on water security. Future studies should therefore investigate how to mitigate unsafe water storage practices in communities without continuous water supply, as well as the long-term sustainability of interventions. Results from this study and future work to understand the health impacts of this intervention will aid stakeholders in optimizing their investments and planning future interventions to improve water quality and access in similar contexts.

## Methods

### Study site and overview

In Mozambique, access to water services is increasing, though unevenly, and rapid urbanization of major cities is introducing new challenges to expanding coverage. In 2022, 87% of residents in Mozambique had at least basic water access [40]. Among this population, 64% of residents reported water being available when needed – though service was intermittent - and accessible on premises. There are disparities in safe drinking water coverage between the wealthiest and poorest residents in Mozambique [20], exacerbating inequalities in the burden of infectious disease, stunted growth, and limited economic development, particularly among women and girls [41]. National data from Mozambique do not include any water quality assessment, so there is no estimate of the proportion of the population that has access to safely managed water. In the city of Beira, our study site, rapid urbanization is straining the existing water infrastructure, and the establishment of informal settlements has created challenges in engineering the expansion of the water supply [42, 43].

In 2016, the World Bank invested $140 million in the public water utility FIPAG (Fundo de Investimento e Património de Abastecimento de ÁAgua) and the regulatory body for water systems, AURA, IP (Autoridade Reguladora de Água, Instituto Publico) as part of the Water Service & Institutional Support (WASIS-II) project to improve water supply in five Mozambican cities [44]. In Beira, the funds were used to construct a new piped water network, increase the number of household water connections, and improve system-wide distribution. In addition to the funds from WASIS-II, the Dutch government provided supplemental funding for the infrastructural upgrades [45].

The PAASIM study *(Pesquisa sobre o Acesso á Água e a Saúde infantii em Moçambique-* Research on Access to Water and Child Health in Mozambique) is a matched control trial designed to study the effects of these piped water supply improvements on child health. PAASIM employs a cluster-matched cohort design, enrolling pregnant mothers in both intervention sub-neighborhoods *(i.e.*, sub-neighborhoods that have received the improvements to the piped water network) and comparison sub-neighborhoods *(i.e.*, sub-neighborhoods that have not yet received improvements) and following their children from birth until 12 months of age [21]. The overall objective of the PAASIM study was to understand the impact of community-level water system improvements on drinking water quality and access, child gut health, diarrhea and growth. The analysis presented here on household drinking water quality comprises one of the pre-specified primary outcomes for the PAASIM study, as well as secondary exposure outcomes. Other primary outcomes *(i.e.*, enteropathogen infection) will be reported elsewhere.

### Data sources

In sub-neighborhoods selected for the study, women in their third trimester of pregnancy were identified and prospectively enrolled and followed until their child was 12 months of age. Eligibility criteria for the women included: 1) 18 years or older, 2) in third trimester of pregnancy, 3) lived in a sub-neighborhood that is targeted for water improvements, or a matched sub-neighborhood not yet targeted for water improvements, 4) not planning to move within the following 12 months, 5) carrying a singleton birth, and 6) consented to take part in the study.

Between February 2021 and October 2023, 642 households in sub-neighborhoods that received water system improvements (intervention) and comparison sub-neighborhoods that did not yet receive the improvements completed the study. We recruited pregnant women via our 2020 population-based survey [20], lists of pregnant women visiting local health centers, and by study staff visits to under-enrolled sub-neighborhoods. We completed five household visits that corresponded with the enrollment timepoint during the mother’s third trimester of pregnancy, and child ages 3, 6, 9 and 12 months. Stored and source water samples were collected from enrolled households at each timepoint throughout the study. Water access data were derived from household surveys conducted at each of the five timepoints. Households were lost to follow-up if participants withdrew from the study, were unreachable, moved out of the study area, had non-singleton births, experienced pregnancy loss, or if the mother or child died. Additional details on sample collection are provided elsewhere (https://osf.io/697vm/).

### Intervention classification

**Figures 4:**
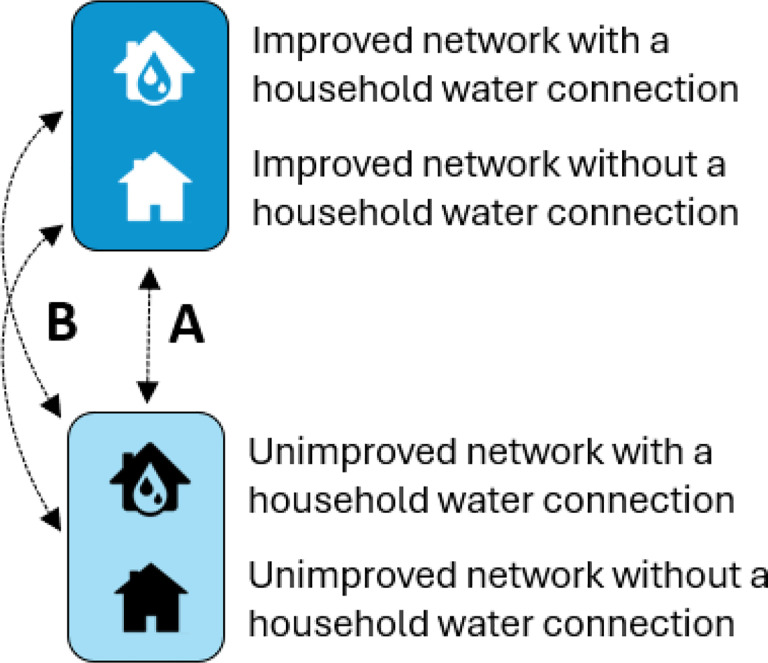
Intervention classifications. **A.** Compares households in neighborhoods which have received the piped water service upgrades to households in comparison neighborhoods. **B.** Compares households which have an active, direct connection to the piped water network to households which do not have an active direct connection to the piped water network, irrespective of neighborhood.

We classified the exposure of interest in two different ways. First, we estimate the impact of living in a sub-neighborhood with an improved piped water network on measures of water quality and access (Fig. 4). Households which moved to a different intervention arm but remained in the study were excluded entirely from the analysis of the impact of the intervention. However, if participants moved within the study area and did not switch intervention arms, they were included in the analysis. We assessed the fidelity of the intervention by estimating the impact of the intervention on the prevalence of direct connections. Second, we estimated the association between having a direct connection to the piped water network and measures of water quality and access (Fig. 4), controlling for intervention status. Each household was assigned a direct connection status at each timepoint.

These separate analyses of the impact of the intervention and association with having a direct connection take into consideration that not all individuals living in intervention sub-neighborhoods are connected to the improved centralized water system at their household and, additionally, that some individuals living in comparison sub-neighborhoods have a direct connection to the (unimproved) water delivery system. We also assess the question of whether there is a difference in effect for households in intervention sub-neighborhoods which have a direct connection (Household Type 1, Fig. 4) compared to those in a comparison sub-neighborhood with no direct connection (Household Type 4, Fig. 4). We measure this by including an interaction term between the intervention status and direct connection status variables to contrast and estimate the effects of having both of these interventions together.

### Water quality and access variables

The pre-specified primary outcome of interest was the prevalence of any *E. coli* in household source water samples at the 12-month timepoint. All stored and source water samples were analyzed for the presence of *E. coli* using the IDEXX Colilert-18 test [46]. The lower limit of detection for the IDEXX Colilert-18 assay was 1 MPN/100 mL, while the upper limit of detection was 2419.6 MPN/100 mL. Secondary outcomes include prevalence of *E. coli* in source and stored water at all timepoints, household access to at least basic water as defined by the Joint Monitoring Programme (JMP) [22], the presence of water that is accessible on the premises of the household or compound, whether households reported having sufficient quantities of drinking water when needed, water insecurity calculated using the Household Water Insecurity Experiences (HWISE) index [47], and satisfaction with water service, affordability, availability, pressure, appearance, and smell. We also assessed the prevalence of households which had source water levels of free chlorine of at least 0.2 mg/L, the minimum standard in the WHO drinking water quality guidelines [48]. Additional details on these variables can be found in Supplemental Table 6. We will also report these secondary outcomes at the 12-month timepoint alone in the supplemental material.

### Additional covariates

Per our study protocol, we controlled for pre-specified confounding variables including: socioeconomic status (SES) defined by the prevalence of high poverty using Mozambique’s Simple Poverty Scorecard [49], observed access to at least basic sanitation defined by the JMP sanitation ladder [50], and secondary education status of the primary caregiver. We analyzed additional variables at the time of enrollment and controlled for any variables that were imbalanced between study arms, including: household density, number of children under age five living in the household, months of residence in the household, presence of human feces in or near the household, presence of animal feces in or near the household, food insecurity measured by the Household Food Insecurity Access Scale (HFIAS) [51], handwashing station in the dwelling or yard, seasonal (rainy vs dry), and any flooding in the household or yard in the past month. Additional details about the covariates and pre-specification of control variables can be found in the pre-specified analysis plan for the PAASIM study (https://osf.io/697vm/).

### Power and sample size

The sample size obtained via the power analyses for the primary study outcome of the PAASIM study (non-viral enteropathogen prevalence in children) [21], providing a power of 80% to detect a relative risk of 0.74 in the primary study outcome. The resulting design yielded a sample size of 642 households followed for 5 visits, for a total of 2,813 source water samples. For our primary exposure outcome of detectible *E. coli* at 12 months in source water, we were thus powered for a minimal detectible effect (MDE) of 7.4 percentage points given an 11.4% prevalence among comparison households at baseline and a calculated intra-class coefficient (ICC) of 0.047. The final ICC at 12-months was 0.001.

### Research Questions

We assessed four research questions: (Q1) What is the impact of the intervention on and association between having a direct connection and prevalence of any *E. coli* in source water (presence versus absence) at the final study visit (when the children were 12 months old) [pre-specified primary outcome of the PAASIM study]?; (Q2) what is the impact of the intervention on water quality and access across all timepoints [pre-specified secondary outcomes]?; (Q3) What is the association between having a direct connection and water quality and access across all timepoints [pre-specified secondary outcomes]?; and (Q4) what is the joint effect of living in intervention sub-neighborhoods and having a direct connection on water quality and access throughout the study period? We hypothesized that individuals who live in sub-neighborhoods where improvements to the piped water network have been made and individuals who have a direct connection to the improved water supply will have better water quality and increased access compared to individuals who do not live in such sub-neighborhoods or households.

### Statistical analysis

All statistical analyses were conducted using R statistical software (RStudio v. 2024.04.2 + 764) and were pre-specified in the analysis plan (https://osf.io/697vm/). We analyzed differences in the outcomes of interest, adjusting for distributions of covariates of interest, between intervention and comparison households for both the intervention and direct connection analyses.

Intervention (Q1 and Q2). We used an approach similar to an intent-to-treat (ITT) analysis to assess the impact of the intervention on water quality and access, where we compare households in intervention sub-neighborhoods to households in comparison sub-neighborhoods, without regard to intervention uptake. We also assessed the fidelity of the intervention by modeling the impact of living in intervention sub-neighborhoods on the prevalence of having a direct connection at the end of our follow-up period. To begin, we fit multivariable log binomial regression models with an independent correlation structure to estimate the prevalence ratio. We also considered fit based on an exchangeable correlation structure using generalized estimating equations (GEE), to account for clustering at the sub-neighborhood level (R package ‘gee’) [52] but the data were not robust enough to support the more complicated model. To ensure our approach was robust, we explored various working covariances in our blinded analysis and found no appreciable changes in the model results. In all adjusted intervention models, we controlled for pre-specified covariates including household SES score at enrollment, observation of at least basic sanitation access at enrollment, and education of the primary caregiver. We adjusted for any potential confounding variables listed above (“Additional covariates”) that were imbalanced at the enrollment timepoint.

The prevalence ratio (PR) was calculated using modified Poisson regression for any log-binomial models that did not converge. Households in the intervention and comparison sub-neighborhoods were group-matched on sub-neighborhood level SES and population density. An indicator variable for matching strata was included in all models.

Direct connection (Q1 and Q3). We used a similar modeling approach to assess the association between having a direct connection and water quality and access, comparing households with an active direct connection to the unimproved piped water network to households without an active direct connection, on the prevalence of *E. coli* in source water at end of follow-up (primary outcome) and on all water quality and access variables throughout the study period (secondary outcomes). We utilized log-binomial models with GEE and an independent correlation structure and fit a modified Poisson model when convergence was not achieved. In addition to controlling for the matching indicator variable, household SES score at enrollment, observation of at least basic sanitation access at enrollment, and education of the primary caregiver, we controlled for the sub-neighborhood intervention status in all models.

Joint effect between intervention and direct connection (Q4). To assess the impact of both living in an intervention sub-neighborhood and having a direct connection, we utilized the same modeling approach described above, adding an interaction term between the two variables which indicate sub-neighborhood intervention and direct connection status. We report stratified model results from any models where the interaction term was significant at an alpha level of 0.05.

### Blinding

Due to the nature of the intervention, participants were not blinded to their intervention status. The study team was blinded to the sub-neighborhood intervention status of each household for the duration of enrollment and sample collection. Unblinding occurred when the analysis of the primary study outcomes was completed by two independent analysts on the study team (https://osf.io/697vm/).

## Figures and Tables

**Table 1:** Characteristics of study participants at enrollment

	Intervention^a^ (N = 302)	Comparison^a^ (N = 309)	Direct connection^a^ (N = 231)	No direct connection^a^ (N = 411)
Number of children under age 5 in household^b^	0.75 (0.8)	0.71 (0.8)	0.71 (0.8)	0.73 (0.7)
Number of people living in household	5.04 (2.4)	4.79 (2.4)	5.7 (2.8)	4.5 (2.0)
Months living in household	78.97 (99.6)	57.46 (73.2)	87 (103.9)	57 (75.2)
Household Food Insecurity Access Scale^c^	9.97 (6.7)	10.04 (7.2)	9.4 (6.6)	10 (7.1)
High poverty^d^	138 (45.7%)	149 (48.2%)	77 (33.3%)	221 (53.8%)
Basic sanitation access^e^	112 (37.2%)	107 (34.7%)	116 (50.7%)	111 (27.0%)
Primary wage earner has fixed employment	95 (33.7%)	127 (44.3%)	101 (45.5%)	129 (34.3%)
Primary caregiver completed secondary education	76 (25.2%)	56 (18.2%)	84 (36.5%)	58 (14.1%)
Human feces observed in or near the household	3 (1.0%)	2 (0.7%)	2 (0.9%)	3 (0.7%)
Animal feces observed in or near the household	35 (11.6%)	26 (8.4%)	29 (12.6%)	33 (8.0%)
Handwashing station (with soap and water) in house or yard^f^	103 (34.1%)	77 (24.9%)	97 (42.0%)	94 (22.9%)
Rainy season (Dec-Apr)	154 (51.0%)	151 (48.9%)	113 (48.9%)	209 (50.9%)
Any flooding in the household or yard in the last month	92 (30.6%)	104 (34.0%)	58 (25.2%)	148 (36.3%)

Data are either mean (SD) or n (%).

a There were 31 households that moved into a neighborhood with a different intervention arm but remained in the study. These households were excluded from the impact of the intervention analysis but remained in the direct connection analysis.

b Because this question was asked of pregnant women at the enrollment timepoint, it is reasonable that the mean number of children under five in the household would be less than 1.

c The Household Food Insecurity Access Scale is calculated using a standardized questionnaire which includes 9 questions that distinguish food insecurity. Higher scores indicate greater food insecurity. [36]

d High poverty is defined by a simple poverty scorecard [34] score of less than or equal to 66.

e Basic sanitation access is defined by having access to improved facilities which are not shared with other households [35]

f We will not control for imbalances in handwashing station with soap or dwelling in the house or yard in the direct connection models as this is on the causal pathway between having a household connection and the water quality and access variables.

**Table 2:** The impact of the intervention on primary and additional water quality and access outcomes. All variables self-reported other than *E. coli* in source and stored water and free chlorine, which were measured by enumerators.^a^

Primary outcome^c^	Intervention (N = 302)^b^	Comparison (N = 309)^b^	Adjusted Prevalence Ratio (95% CI)
E *coli* in source water (12-month visit)	37 (13%)	50 (20%)	0.67 (0.46, 0.98)
Secondary outcomes^d^	Intervention (N = 1510)^b^	Comparison (N = 1545)^b^	Adjusted Prevalence Ratio (95% CI)
E *coli* in source water (all visits)	170 (12%)	198 (15%)	0.74 (0.56, 0.97)
E *coli* in stored water (all visits)	652 (44%)	725 (48%)	0.86 (0.77, 0.96)
Water insecure	331 (22%)	247 (16%)	1.09 (0.74, 1.62)
Access to basic water	1474 (98%)	1394 (90%)	1.09 (1.01, 1.17)
Water available on premises	892 (59%)	677 (44%)	1.31 (1.04, 1.64)
Access to sufficient quantities of water	1296 (86%)	1346 (87%)	1.03 (0.98, 1.07)
Always satisfied with water service	899 (60%)	653 (42%)	1.37 (1.16, 1.62)
Always satisfied with water affordability	1058 (70%)	899 (59%)	1.21 (1.03, 1.42)
Always satisfied with water availability	951 (63%)	827 (54%)	1.24 (1.05, 1.48)
Always satisfied with water pressure	900 (60%)	805 (53%)	1.15 (0.97, 1.35)
Always satisfied with water appearance	433 (29%)	397 (26%)	1.04 (0.80, 1.35)
Always satisfied with water taste and smell	690 (55%)	668 (43%)	0.98 (0.84, 1.14)
Water consumption ≥ 80 L per day	937 (62%)	857 (55%)	1.14 (0.99, 1.30)
Free chlorine > = 0.2 mg/L in source water	889 (65%)	795 (62%)	0.96 (0.86, 1.07)

Data are n (%).

a In all intervention models, we controlled for months living in household and fixed employment of the primary wage earner in addition to the pre-specified covariates.

b These numbers indicate the total number of household observations across all five visits in each of the study households throughout the study period.

c Pre-specified primary outcome was the prevalence of *E. coli* in source water at the end of follow-up which corresponds with the 12-month visit.

d Pre-specified secondary outcomes were all the water quality and access measures across all five timepoints throughout the study.

**Table 3:** The association between having a direct connection and primary and additional water quality and access outcomes. All variables self-reported other than *E. coli* in source and stored water and free chlorine, which were measured by enumerators.^a^

Primary outcome^c^	Direct connection (N = 262)^b^	No direct connection (N = 380)^b^	Adjusted Prevalence Ratio (95% CI)
E *coli* in source water (12-month visit)	37 (15%)	54 (18%)	0.76 (0.53, 1.10)
Secondary outcomes^d^	Direct connection (N = 1244)^b^	No direct connection (N = 1965)^b^	Adjusted Prevalence Ratio (95% CI)
E *coli* in source water (all visits)	142 (12%)	241 (15%)	0.80 (0.59, 1.08)
E *coli* in stored water (all visits)	546 (45%)	896 (46%)	1.00 (0.89, 1.11)
Water insecure	293 (24%)	319 (16%)	1.27 (1.03, 1.58)
Access to basic water	1222 (98%)	1792 (91%)	1.04 (1.01, 1.07)
Water available on premises	1195 (96%)	467 (24%)	3.79 (3.07, 4.69)
Access to sufficient quantities of water	1096 (88%)	1680 (86%)	1.00 (0.95, 1.04)
Always satisfied with water service	732 (59%)	910 (46%)	1.19 (1.04, 1.36)
Always satisfied with water affordability	953 (77%)	1108 (57%)	1.31 (1.17, 1.46)
Always satisfied with water availability	802 (65%)	1068 (54%)	1.11 (0.99, 1.25)
Always satisfied with water pressure	771 (63%)	1027 (53%)	1.15 (1.02, 1.29)
Always satisfied with water appearance	371 (30%)	500 (25%)	1.15 (0.97, 1.38)
Always satisfied with water taste and smell	559 (45%)	866 (44%)	1.00 (0.88, 1.13)
Water consumption > = 80 L per day	848 (68%)	1034 (53%)	1.31 (1.13, 1.53)
Free chlorine > = 0.2 mg/L in source water	771 (66%)	1002 (62%)	1.13 (1.02, 1.26)

Data are in n (%).

a In all direct connection models, we controlled for months living in the household, fixed employment of the primary wage earner, and any flooding in the household or yard in the past month in addition to the pre-specified covariates (see Methods).

b These numbers indicate the total number of household observations across all five visits in each of the study households throughout the study period.

c Our pre-specified primary outcome was the prevalence of *E. coli* in source water at the end of follow-up which corresponds with the 12-month visit.

d Our pre-specified secondary outcomes were all the water quality and access measures across all five timepoints throughout the study.

## References

[1] Clasen TF, Alexander KT, Sinclair D, Boisson S, Peletz R, Chang HH, Interventions to improve water quality for preventing diarrhoea. Cochrane Database Syst Rev. 2015 Oct 20;2015(10):CD004794. 26488938 10.1002/14651858.CD004794.pub3 PMC4625648

[2] Wolf J, Hunter PR, Freeman MC, Cumming O, Clasen T, Bartram J, Impact of drinking water, sanitation and handwashing with soap on childhood diarrhoeal disease: updated meta-analysis and meta-regression. Trop Med Int Health. 2018 May;23(5):508–25. 29537671 10.1111/tmi.13051

[3] Wolf J, Johnston RB, Ambelu A, Arnold BF, Bain R, Brauer M, Burden of disease attributable to unsafe drinking water, sanitation, and hygiene in domestic settings: a global analysis for selected adverse health outcomes. The Lancet. 2023 Jun 17;401(10393):2060–71. 10.1016/S0140-6736(23)00458-0 PMC10290941 37290458

[4] WHO/UNICEF Joint Monitoring Programme for Water Supply, Sanitation and Hygiene. Progress on household drinking water, sanitation, and hygiene: 2000–2022 [Internet]. 2023 Jul. Available from: https://washdata.org/reports/jmp-2023-wash-households-gender-pullout-launch

[5] UN-Water. UN-Water GLAAS 2019: National systems to support drinking-water, sanitation and hygiene - Global status report 2019 [Internet]. 2019 [cited 2021 Oct 1]. Available from: https://www.unwater.org/publications/un-water-glaas-2019-national-systems-to-support-drinking-water-sanitation-and-hygiene-global-status-report-2019/

[6] Brown J, Hien VT, McMahan L, Jenkins MW, Thie L, Liang K, Relative benefits of on-plot water supply over other “improved” sources in rural Vietnam. Trop Med Int Health. 2013 Jan;18(1):65–74. 23107456 10.1111/tmi.12010

[7] Chongsuvivatwong V, Mo-suwan L, Chompikul J, Vitsupakorn K, McNeil D. Effects of piped water supply on the incidence of diarrheal diseases in children in southern Thailand. Southeast Asian J Trop Med Public Health. 1994 Dec;25(4):628–32. 7667703

[8] Devoto F, Duflo E, Dupas P, Parienté W, Pons V. Happiness on Tap: Piped Water Adoption in Urban Morocco. American Economic Journal: Economic Policy. 2012 May;4(4):68–99.

[9] Ercumen A, Arnold BF, Kumpel E, Burt Z, Ray I, Nelson K, Upgrading a piped water supply from intermittent to continuous delivery and association with waterborne illness: a matched cohort study in urban India. PLoS Med. 2015 Oct;12(10):e1001892. 26505897 10.1371/journal.pmed.1001892 PMC4624240

[10] Water Expansions in Shantytowns: Health and Savings - GALIANI - 2009 - Economica - Wiley Online Library [Internet]. [cited 2024 Sep 16]. Available from: https://onlinelibrary.wiley.com/doi/abs/10.1111/j.1468-0335.2008.00719.x

[11] Hunter PR, Ramírez Toro GI, Minnigh HA. Impact on diarrhoeal illness of a community educational intervention to improve drinking water quality in rural communities in Puerto Rico. BMC Public Health. 2010 Apr 28;10:219. 20426831 10.1186/1471-2458-10-219 PMC2876105

[12] Klasen S, Lechtenfeld T, Meier K, Rieckmann J. Benefits trickling away: The health impact of extending access to piped water and sanitation in urban Yemen [Internet]. Discussion Papers; 2012 [cited 2024 Sep 16]. Report No.: 110. Available from: https://www.econstor.eu/handle/10419/90441

[13] Pickering AJ, Crider Y, Sultana S, Swarthout J, Goddard FG, Anjerul Islam S, Effect of in-line drinking water chlorination at the point of collection on child diarrhoea in urban Bangladesh: a double-blind, cluster-randomised controlled trial. Lancet Glob Health. 2019 Sep;7(9):e1247–56. 31402005 10.1016/S2214-109X(19)30315-8

[14] Reese H, Routray P, Torondel B, Sinharoy SS, Mishra S, Freeman MC, Assessing longer-term effectiveness of a combined household-level piped water and sanitation intervention on child diarrhoea, acute respiratory infection, soil-transmitted helminth infection and nutritional status: a matched cohort study in rural Odisha, India. Int J Epidemiol. 2019 Dec 1;48(6):1757–67. 31363748 10.1093/ije/dyz157 PMC6929523

[15] Ryder RW, Reeves WC, Singh N, Hall CB, Kapikian AZ, Gomez B, The childhood health effects of an improved water supply system on a remote Panamanian island. American Journal of Tropical Medicine & Hygiene. 1985 Sep;34(5):921–4. 4037183 10.4269/ajtmh.1985.34.921

[16] Wang ZS, Shepard DS, Zhu YC, Cash RA, Zhao RJ, Zhu ZX, Reduction of enteric infectious disease in rural China by providing deep-well tap water. Bulletin of the World Health Organization. 1989;67(2):171–80. 2501042 PMC2491231

[17] Wolf J, Hubbard S, Brauer M, Ambelu A, Arnold BF, Bain R, Effectiveness of interventions to improve drinking water, sanitation, and handwashing with soap on risk of diarrhoeal disease in children in low-income and middle-income settings: a systematic review and meta-analysis. Lancet. 2022 Jul 2;400(10345):27–40. 10.1016/S0140-6736(22)00937-0 PMC9251635 35780792

[18] Goddard F, Ban R, Barr DB, Brown J, Cannon J, Colford JM, Measuring environmental exposure to enteric pathogens in low-income settings: Review and recommendations of an interdisciplinary working group. Environmental Science & Technology [Internet]. 2020 Aug 19 [cited 2020 Aug 27]; Available from: https://pubs.acs.org/doi/pdf/10.1021/acs.est.0c02421 10.1021/acs.est.0c02421 PMC7547864 32813503

[19] Tincani L, Ross I, Zaman R, Burr P, Mujica A, Ensink J, Regional assessment of the operational sustainability of water and sanitation services in Sub-Saharan Africa [Internet]. VFM-WASH; 2015. Report No.: 10.17037/PUBS.04648765. Available from: https://researchonline.lshtm.ac.uk/id/eprint/4648765/

[20] Victor C, Ocasio DV, Cumbe ZA, Garn JV, Hubbard S, Mangamela M, Spatial Heterogeneity of Neighborhood-Level Water and Sanitation Access in Informal Urban Settlements: A Cross-Sectional Case Study in Beira, Mozambique. Accepted: PLOS Water. 2022 Jan 27;2022.01.25.22269649. 10.1371/journal.pwat.0000022 PMC9573900 36258753

[21] Levy K, Garn JV, Cumbe ZA, Muneme B, Fagnant-Sperati CS, Hubbard S, Study design and rationale for the PAASIM project: a matched cohort study on urban water supply improvements and infant enteric pathogen infection, gut microbiome development and health in Mozambique. BMJ Open. 2023 Mar 1;13(3):e067341. 10.1136/bmjopen-2022-067341 PMC9990653 36863743

[22] Drinking water | JMP [Internet]. [cited 2022 Mar 31]. Available from: https://washdata.org/monitoring/drinking-water

[23] Bain R, Johnston R, Khan S, Hancioglu A, Slaymaker T. Monitoring Drinking Water Quality in Nationally Representative Household Surveys in Low- and Middle-Income Countries: Cross-Sectional Analysis of 27 Multiple Indicator Cluster Surveys 2014–2020. Environ Health Perspect. 2021 Sep;129(9):97010. 34546076 10.1289/EHP8459 PMC8454503

[24] Shaheed A, Orgill J, Montgomery MA, Jeuland MA, Brown J. Why “improved” water sources are not always safe. Bulletin of the World Health Organization. 2014 Jan 10;92(4):283. 24700996 10.2471/BLT.13.119594 PMC3967570

[25] Bivins A, Lowry S, Wankhede S, Hajare R, Murphy HM, Borchardt M, Microbial water quality improvement associated with transitioning from intermittent to continuous water supply in Nagpur, India. Water Research. 2021 Aug 1;201:117301. 34139512 10.1016/j.watres.2021.117301

[26] Kumpel E, Nelson KL. Mechanisms affecting water quality in an intermittent piped water supply. Environ Sci Technol. 2014;48(5):2766–75. 24459990 10.1021/es405054u

[27] Whittington D, Cook J. Valuing Changes in Time Use in Low- and Middle-Income Countries. J Benefit Cost Anal. 2019;10(Suppl 1):51–72. 32983833 10.1017/bca.2018.21 PMC7473062

[28] Graham JP, Hirai M, Kim S-S. An Analysis of Water Collection Labor among Women and Children in 24 Sub-Saharan African Countries. PLoS One. 2016;11(6):e0155981. 27248494 10.1371/journal.pone.0155981 PMC4889070

[29] Mcgranahan G, Walnycki A, Dominick F, Kombe W, Kyessi A, Limbumba T, How International Water and Sanitation Monitoring Fails Deprived Urban Dwellers. In 2018. p. 117–31.

[30] Zuin V, Ortolano L, Davis J. The entrepreneurship myth in small-scale service provision: Water resale in Maputo, Mozambique. Journal of Water, Sanitation and Hygiene for Development. 2014 Jun 1;4(2):281–92.

[31] Zuin V, Nicholson M, Davis J. Water access, service quality, and consumer satisfaction in peri-urban Maputo: A beneficiary assessment. :77.

[32] Zuin V, Nicholson M, Davis J. Water access, poverty, and policy changes in peri-urban Maputo, Mozambique. :125.

[33] Zuin V, Ortolano L, Alvarinho M, Russel K, Thebo A, Muximpua O, Water supply services for Africa’s urban poor: the role of resale. Journal of Water and Health. 2011 Dec 1;9(4):773–84. 22048436 10.2166/wh.2011.031

[34] Luengo M, Banerjee S, Keener S. Provision Of Water To The Poor In Africa : Experience With Water Standposts And The Informal Water Sector [Internet]. The World Bank; 2010 [cited 2022 Apr 26]. 65 p. (Policy Research Working Papers). Available from: https://elibrary.worldbank.org/doi/abs/10.1596/1813-9450-5387

[35] Huynh L, Anjum S, Lieu T, Horse ML, Martin-Hill D, Wekerle C. Examining the connection between water concerns, water anxiety, and resilience among Indigenous persons: A systematic scoping review. Child Abuse & Neglect. 2024 Feb 1;148:106184. 37055333 10.1016/j.chiabu.2023.106184

[36] Toivettula A, Varis O, Vahala R, Juvakoski A. Making waves: Mental health impacts of inadequate drinking water services — From sidenote to research focus. Water Research. 2023 Sep 1;243:120335. 37516073 10.1016/j.watres.2023.120335

[37] Denantes J, Donoso G. Factors influencing customer satisfaction with water service quality in Chile. Utilities Policy. 2021 Dec 1;73:101295.

[38] Turgeon S, Rodriguez MJ, Theriault M, Levallois P. Perception of drinking water in the Quebec City region (Canada): the influence of water quality and consumer location in the distribution system. Journal of Environmental Management. 2004 Apr 1;70(4):363–73. 15016444 10.1016/j.jenvman.2003.12.014

[39] Lemaitre AK, Miller JD, Stoler J. Household water insecurity experiences and their perceived determinants in a low-income community of Cartagena, Colombia, during a water service expansion project. PLOS Water. 2023 Sep 5;2(9):e0000154.

[40] World Health Organization (WHO) and the United Nation’s Children’s Fund (UNICEF). JMP Global Database [Internet]. [cited 2024 Jun 25]. Available from: https://washdata.org/data/household#!/

[41] rd United Nations World Water Development Report: Water in a Changing World .:. Sustainable Development Knowledge Platform [Internet]. [cited 2022 Mar 31]. Available from: https://sustainabledevelopment.un.org/index.php?page=view&type=400&nr=96&menu=1515

[42] Dagdeviren H, Robertson SA. Access to water in the slums of the developing world [Internet]. Working Paper; 2009 [cited 2022 Mar 31]. Report No.: 57. Available from: https://www.econstor.eu/handle/10419/71784

[43] Dos Santos S, Adams EA, Neville G, Wada Y, de Sherbinin A, Mullin Bernhardt E, Urban growth and water access in sub-Saharan Africa: Progress, challenges, and emerging research directions. Sci Total Environ. 2017 Dec 31;607–608:497–508. 10.1016/j.scitotenv.2017.06.157 28704674

[44] Development Projects : Water Services & Institutional Support II - P149377 [Internet]. World Bank. [cited 2024 Nov 6]. Available from: https://projects.worldbank.org/en/projects-operations/project-detail/P149377

[45] Netherlands M of FA of the K of the. Dutch Development results Mozambique [Internet]. [cited 2022 Apr 20]. Available from: https://www.dutchdevelopmentresults.nl/2019/countries/mozambique#water

[46] Sercu B, Van De Werfhorst LC, Murray JLS, Holden PA. Cultivation-independent analysis of bacteria in IDEXX Quanti-Tray/2000 fecal indicator assays. Appl Environ Microbiol. 2011 Jan;77(2):627–33. 21097584 10.1128/AEM.01113-10 PMC3020531

[47] Young SL, Boateng GO, Jamaluddine Z, Miller JD, Frongillo EA, Neilands TB, The Household Water InSecurity Experiences (HWISE) Scale: development and validation of a household water insecurity measure for low-income and middle-income countries. BMJ Glob Health. 2019;4(5):e001750. 10.1136/bmjgh-2019-001750 PMC6768340 31637027

[48] World Health Organization, editor. Guidelines for drinking-water quality. 4th ed. Geneva: World Health Organization; 2011. 541 p.

[49] Schreiner M. Simple Poverty Scorecard^®^ Poverty-Assessment Tool Mozambique. :127.

[50] Sanitation | JMP [Internet]. [cited 2024 Nov 11]. Available from: https://washdata.org/monitoring/sanitation

[51] Coates J, Swindale A, Bilinsky P. Household Food Insecurity Access Scale (HFIAS) for Measurement of Food Access: Indicator Guide: Version 3: (576842013–001) [Internet]. American Psychological Association; 2007 [cited 2022 Oct 31]. Available from: http://doi.apa.org/get-pe-doi.cfm?doi=10.1037/e576842013-001

[52] Carey V. gee: Generalized Estimation Equation Solver [Internet]. Available from: https://CRAN.R-project.org/package=gee

